# AnnCovDB: a manually curated annotation database for mutations in SARS-CoV-2 spike protein

**DOI:** 10.1093/database/baaf002

**Published:** 2025-02-12

**Authors:** Xiaomin Zhang, Zhongyi Lei, Jiarong Zhang, Tingting Yang, Xian Liu, Jiguo Xue, Ming Ni

**Affiliations:** Academy of Military Medical Sciences, No. 27 Taiping Road, Haidian District, Beijing 100850, PR China; Academy of Military Medical Sciences, No. 27 Taiping Road, Haidian District, Beijing 100850, PR China; College of Life Science and Technology, Beijing University of Chemical Technology, No.15 North Third Ring Road East, Chaoyang District, Beijing 100029, PR China; Academy of Military Medical Sciences, No. 27 Taiping Road, Haidian District, Beijing 100850, PR China; School of Forensic Medicine, Shanxi Medical University, No.98, University Street, Wujinshan Town, Yuci District, Jinzhong, Shanxi Province 030600, PR China; Academy of Military Medical Sciences, No. 27 Taiping Road, Haidian District, Beijing 100850, PR China; School of Forensic Medicine, Shanxi Medical University, No.98, University Street, Wujinshan Town, Yuci District, Jinzhong, Shanxi Province 030600, PR China; Academy of Military Medical Sciences, No. 27 Taiping Road, Haidian District, Beijing 100850, PR China; Academy of Military Medical Sciences, No. 27 Taiping Road, Haidian District, Beijing 100850, PR China; Academy of Military Medical Sciences, No. 27 Taiping Road, Haidian District, Beijing 100850, PR China

## Abstract

Severe acute respiratory syndrome coronavirus 2 (SARS-CoV-2) has been circulating and adapting within the human population for >4 years. A large number of mutations have occurred in the viral genome, resulting in significant variants known as variants of concern (VOCs) and variants of interest (VOIs). The spike (S) protein harbors many of the characteristic mutations of VOCs and VOIs, and significant efforts have been made to explore functional effects of the mutations in the S protein, which can cause or contribute to viral infection, transmission, immune evasion, pathogenicity, and illness severity. However, the knowledge and understanding are dispersed throughout various publications, and there is a lack of a well-structured database for functional annotation that is based on manual curation. AnnCovDB is a database that provides manually curated functional annotations for mutations in the S protein of SARS-CoV-2. Mutations in the S protein carried by at least 8000 variants in the GISAID were chosen, and the mutations were then utilized as query keywords to search in the PubMed database. The searched publications revealed that 2093 annotation entities for 205 single mutations and 93 multiple mutations were manually curated. These entities were organized into multilevel hierarchical categories for user convenience. For example, one annotation entity of N501Y mutation was ‘Infectious cycle➔Attachment➔ACE2 binding affinity➔Increase’. AnnCovDB can be used to query specific mutations and browse through function annotation entities.

**Database URL**: https://AnnCovDB.app.bio-it.tech/

## Introduction

By June 2024, the number of coronavirus disease 2019 (COVID-19) cases and deaths reported to the World Health Organization was over 775 million and caused 7 million [1]. The COVID-19 viral pathogen, severe acute respiratory syndrome coronavirus 2 (SARS-CoV-2), has highly diverse genomes and has evolved into about 2000 lineages [2]. Specifically, the variants of the Omicron lineage have dramatically more mutations in the spike (S) protein compared to those of previous variants of concern [3]. The circulation and adaption of the Omicron variants in the human population is continuing, resulting in thousands of deaths worldwide each month [1].

The S protein of SARS-CoV-2 binds to the angiotensin-converting enzyme 2 (ACE2) receptor on the surface of human cells. This binding initiates the entry of viral particles into the cell through TMPRSS2 or other endosomal proteases, which are associated with the non-Omicron and Omicron variants, respectively [4–7]. The S protein is essential for SARS-CoV-2, and mutations in the protein that improve SARS-CoV-2 fitness occur continuously [8]. For example, the D614G mutation in the S protein emerged in February 2020, preventing premature S1 shedding and thereby increasing infectivity [9–13]. N501Y significantly increases the affinity of the S protein for ACE2, which is amplified by epistasis interaction with the Q498R mutation [14–17]. The P681R mutation in the furin cleavage site of the Delta variant increased fusogenicity and pathogenicity [18–20]. Many mutations, including E484K/Q and L452R, have been shown to confer immune evasion capabilities, particularly for the Omicron variants [21, 22].

While many studies have been focused on the functions of S protein mutations, there is currently a paucity of databases that encompass relevant publications and provide a thorough integration of functional annotations. The COV2Var database, developed by Feng *et al*., provides a comprehensive computational assessment of the effects of SARS-CoV-2 mutations and their relationships with various factors [23]; however, publications queried by specific mutations are shown automatically, without manual curation. The CoVariants (https://covariants.org) database and the Stanford Coronavirus Resistance Database (CoV-RDB) both provide manually curated annotations for mutations in the S protein of SARS-CoV-2; whereas, CoVariants only includes 12 mutations, and CoV-RDB focuses on neutralizing susceptibility to monoclonal antibodies, convalescent plasma, and vaccine plasma [24]. Besides, a database of human viruses transmitted via aerosols named AVM includes the immune escape of SARS-CoV-2 [25]. In 2023, Song *et al*. developed a comprehensive database named RCoV19, which included 12 554 entries about the mutations in the SARS-CoV-2 genome, assigned to six major aspects such as “infectivity/transmissibility,” “antibody resistant,” and “drug resistant” [26]. Despite all these databases, more detailed functional annotations about the mutations in the S protein of SARS-CoV-2 are still needed.

Here we presented an integrated and convenient query for annotations of mutations in the S protein of SARS-CoV-2. Based on 717 selected publications, we manually annotated the functions and/or effects of 205 single mutations and 93 multiple mutations in the S protein. We organized annotation entities using multilevel hierarchical categories and developed AnnCovDB, an annotation database for mutations in the S protein, with a searching and viewing interface.

## Materials and methods

### Data collection

**Figure 1.**
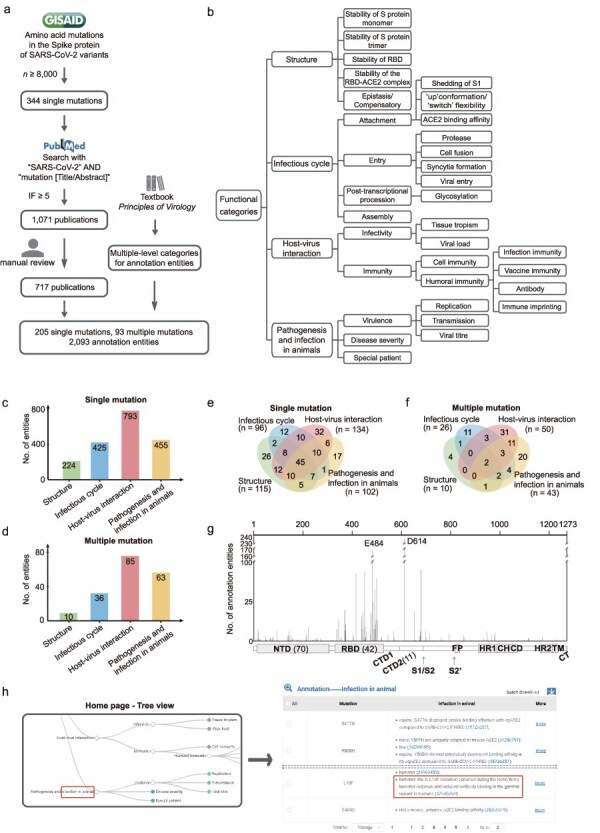
Overview of AnnCovDB. (a) A scheme for selecting mutations and publications for manual annotation curation. (b) Multiple-level hierarchical categories for organizing annotated entities of mutations. (c–d) The numbers of annotation entities of the four top-level categories for single mutations (c) and multiple mutations (d). (e–f) Venn diagrams of annotation entities divided into the four top-level categories for the single mutations (e) and multiple mutations (f). (g) The number of annotation entities in mutations located in the spike protein. The number of mutations in specific domains is listed in the brackets. (h) Screenshots of AnnCovDB pages for the browsing mutations associated with infection in animal.

Figure 1a shows a scheme for selecting amino acid mutations in the S protein of SARS-CoV-2 and searching for relevant publications. On 25 January 2024, we downloaded the dataset of amino acid mutations in the S protein involving 16 419 647 SARS-CoV-2 variants from the GISAID database (https://gisaid.org), using the wild-type sequence (Wuhan-hu-1 strain, GISAID accession EPI_ISL_402125) as reference. We selected mutations that are harbored in the S protein of at least 8000 SARS-CoV-2 variants. Then, on 8 November 2024, relevant publications in the PubMed database were searched using the keywords “SARS-CoV-2” AND “mutation [Title/Abstract].” The papers were filtered for journals with a ≥ 5 impact factor, obtaining 1071 publications. These publications were reviewed for descriptions of the functions or effects of S protein mutations, and annotation entities were manually curated based on 717 of them. If the conclusions of two publications on one aspect of a mutation differ, we reserve both. The data collection is performed every 3 months to include new publications for mutation annotation.

### Multiple-level hierarchical organization of functional annotation entities

A large proportion of mutations contain multiple entities for the functional annotations. To categorize them, we introduce multilevel hierarchical categories, which are primarily adopted from the textbook “Principles of Virology” [27, 28]. The categories are divided into four levels of categories, as shown in Fig. 1b. The top-level categories are “Structure,” “Infectious cycle,” “Host–virus interaction,” and “Pathogenesis and infection in animals.” The “Structure” category includes the impact of mutations on the three-dimensional structure of the S protein as well as the stability. The “Infectious cycle” category records how mutations in the S protein affect SARS-CoV-2’s attachment, entry, post-transcriptional, and assembly procession. The “Host–virus interaction” is divided into sub-categories “infectivity” and “immunity,” including entities such as the effect of mutations on the immune escape of monoclonal antibodies and convalescent plasma. The “Pathogenesis” in “Pathogenesis and infection in animals” records the clinical manifestations of SARS-CoV-2 infection in humans. Considering the important role of animals in COVID-19 as natural survivors of SARS-CoV-2 [29, 30], we include “Infection in animal” in the 4th top category to record the viral mutations that occurred in animals and/or led to animal infections.

### Web interface implementation

The front-end interface of the AnnCovDB was developed with JavaScript, a progressive JavaScript framework, and styled using Element UI (https://element.eleme.cn/) and Vuetify (https://vuetifyjs.com/en/), which provide a rich collection of user-interface components. Data visualization is implemented with ECharts (https://echarts.apache.org/en/). On the back-end, Node.js (https://nodejs.org/en), a JavaScript runtime built on Chrome’s V8 JavaScript engine, powers the server-side logic, while MongoDB serves (https://www.mongodb.com/) as the database for storing application data. For deployment, NGINX (https://nginx.org/en/) is used to serve the application to users efficiently. The AnnCovDB is freely available at https://AnnCovDB.app.bio-it.tech/.

## Results

### Data statistics

Based on 717 publications, functional annotations for 205 single mutations and 93 multiple mutations in SARS-CoV-2 S protein were manually curated, generating a total of 2093 annotation entities. These entities were organized hierarchically into four-level categories (Fig. 1b). As shown in Fig. 1c and d, the top-level category “Host–virus interaction” has the most annotation entities, followed by “Pathogenesis and infection in animals,” “Infectious cycle,” and “Structure.” About half of the single or multiple mutations (146 of 298, 49.0%) had more than one annotation category, and many of them were dispersed across multiple top-level categories, referred to as polyfunctional mutations. Of the 205 single mutations, 119 (58.0%) were polyfunctional (Fig. 1e); moreover, 45 (22.0%) contained annotation entities from four top-level categories. Notably, 31 of the 34 (91%) characteristic mutations identified in the Omicron BA.1 variants were polyfunctional [31].

For multiple mutations, 29.0% (27 of 93) are polyfunctional (Fig. 1f), with only two falling into four top-level categories (L452R/T478K and L452R/E484Q). Five mutations are prevalent in the combinations of multiple mutations, including D614G (within 33 multiple mutations), N501Y (25), E484K (17), S477N (11), and Q498R (10).

Sixty-seven (32.7%) single mutations are located in the receptor-binding domain (RBD, residues from 331 to 528) of the S protein, and 70 (34.1%) are in the N-terminal domain (NTD, residues from 14 to 306). RBD has 42 (45.2%) multiple mutations, while NTD has only one. As shown in Fig. 1g, the mutations in RBD and the S1/S2 cleavage site had remarkably more annotation entities. D614 and E484 are the two most extensively investigated mutations, with more than 100 annotation entities.

### Applications

#### Searching functional annotations of “P681R” in the furin cleavage site

The characteristic mutation P681R in the S protein of B.1.617 (Delta) variants is well known for significantly increasing S1/S2 cleavage efficiency, resulting in faster viral replication [20]. However, it is less known that P681R is a polyfunctional mutation. Using the AnnCovDB database, users could obtain 45 annotation entities of P681R, which are spread in four top-level categories including “Infectious cycle” (*n* = 20), “Pathogenesis and infection in animals” (*n* = 18), “Host–virus interaction” (*n* = 6), and “Structure” (*n* = 1). For example, in the “Structure” category, P681R was predicted to decrease the stability of the S protein using the deep-learning-based method DeepDDG [32, 33]. In the “Host–virus interaction” category, the variants with P681R/D614G had a lower T-cell immunogenicity than those with simply D614G [34]. The combination of L452R/P681R/D950N had an annotation in “Pathogenesis and infection in animals” that was identified as essential for the higher ACE2 downregulation activity observed in the Delta variant compared to that in the other variants of concern [35].

#### Searching mutations and annotations related to SARS-CoV-2 infection in hamsters

Users could choose the “infection in animals” in the “Pathogenesis and infection in animals” top-level category from the interactive hierarchical categories of entities, and AnnCovDB would yield 40 single mutations and 12 multiple mutations related to this category (Fig. 1h). The annotation entity descriptions included animal species and identified nine mutations associated with SARS-CoV-2 infection in hamsters. H655Y, for example, has been associated with increased efficient transmission in a hamster infection model, possibly through enhanced S cleavage and viral growth [36]. On 14 October 2021, the L18F mutation emerged during the Hong Kong hamster outbreak [37]. Furthermore, neuroinvasion was associated with neuroinflammation in the olfactory bulb of hamsters inoculated with D614G [38].

## Conclusion and discussion

Overall, AnnCovDB is a database of manually curated annotations for mutations in the S protein of SARS-CoV-2 with a high frequency. A total of 2093 annotation entities were organized into multiple-level hierarchical categories for users’ convenience. This database allows researchers to more efficiently search for the functional effects or related underlying mechanisms of SARS-CoV-2 mutations. Additionally, considering the diminishing influence of COVID-19 on people and the subsequent decrease in the volume of new articles, this study plans to refresh the database every 3 months.

In some cases, the functional annotations about one mutation are inconsistent. We found that part could be attributed to the different methodologies. For instance, the mutation S371 F was described as enhancing the ACE2 affinity using the computational docking method [39], while in one deep mutational scanning dataset, it appears to decrease hACE2 affinity [40]. These inconsistencies were all recorded in AnnCovDB.

In the current version, the publications were queried in PubMed databases and filtered with certain criteria. We also tested using an artificial intelligence tool, ChatGPT, for the literature search. However, ChatGPT failed to provide a complete list of relevant publications. For example, for the Y505H mutation, ChatGPT v3.5 gave three publications and provided three annotations including “loss of hydrogen bonding,” “role in immune evasion and infectivity,” and “stability and structural Changes.” In contrast, AnnCovDB contained 26 annotation entities for Y505H based on 24 publications. Recently, Lehr *et al*. reported that ChatGPT is a poor curator of scientific articles [41]. Therefore, manual curation might be labor-intensive but still useful approach. Thus, AnnCovDB uses the retrieval of the PubMed database and manually curated annotation.

The major limitation of AnnCovDB is that the annotations were largely dependent on the curators since AnnCovDB contained 29 categories for annotations. For a comparison, the databases CoV-RDB and RCoV19 have less than six annotation categories and the annotations are more standardized. It is a trade-off between the abundance of categories and standardization. More categories might provide more convenience for users to find the annotations of interest.

## Data Availability

All data in AnnCovDB are freely accessible at https://AnnCovDB.app.bio-it.tech/.
